# Evaluating PCR-Based Detection of *Salmonella* Typhi and Paratyphi A in the Environment as an Enteric Fever Surveillance Tool

**DOI:** 10.4269/ajtmh.18-0428

**Published:** 2018-11-12

**Authors:** Senjuti Saha, Arif M. Tanmoy, Jason R. Andrews, Mohammad S. I. Sajib, Alexander T. Yu, Stephen Baker, Stephen P. Luby, Samir K. Saha

**Affiliations:** 1 Child Health Research Foundation, Department of Microbiology, Dhaka Shishu Hospital, Dhaka, Bangladesh;; 2 Department of Medical Microbiology and Infectious Diseases, Erasmus University Medical Centre, Rotterdam, The Netherlands;; 3 Division of Infectious Diseases and Geographic Medicine, Department of Medicine, Stanford University, Stanford, California;; 4 Enteric Infections, Oxford University Clinical Research Unit, Ho Chi Minh City, Vietnam;; 5 Bangladesh Institute of Child Health, Dhaka Shishu Hospital, Dhaka, Bangladesh

## Abstract

With prequalification of a typhoid conjugate vaccine by the World Health Organization, countries are deciding whether and at what geographic scale to provide the vaccine. Optimal local data to clarify typhoid risk are expensive and often unavailable. To determine whether quantitative polymerase chain reaction (qPCR) can be used as a tool to detect typhoidal *Salmonella* DNA in the environment and approximate the burden of enteric fever, we tested water samples from urban Dhaka, where enteric fever burden is high, and rural Mirzapur, where enteric fever burden is low and sporadic. Sixty-six percent (38/59) of the water sources of Dhaka were contaminated with typhoidal *Salmonella* DNA, in contrast to none of 33 samples of Mirzapur. If these results can be replicated in larger scale in Bangladesh and other enteric fever endemic areas, drinking water testing could become a low-cost approach to determine the presence of typhoidal *Salmonella* in the environment that can, in turn, guide informed-design of blood culture-based surveillance and thus assist policy decisions on investing to control typhoid.

Enteric fever, caused by infection with *Salmonella* Typhi or Paratyphi A, B, or C (typhoidal *Salmonella*), is among the most common bacterial causes of morbidity worldwide, with the greatest burden occurring in low- and middle-income countries (LMICs).^1^ However, estimates of enteric fever incidence suffer from coarse geographical and temporal resolution, because of a lack of surveillance systems for these diseases. This paucity of incidence data is in part because traditional surveillance requires population-based surveillance, which is resource intensive, requiring both robust laboratory infrastructure and population-based clinical data collection with substantial numbers of participants. Consequently, very few LMICs routinely conduct these activities on any scale and estimates of enteric fever burden are largely derived from historical studies, which were generally conducted in high-risk urban settings, such as slums, with results then extrapolated to entire countries or regions. Furthermore, use of antibiotics before seeking care is very common in many countries, which compromises the sensitivity of blood culture.^2^

Robust, low-cost, and sustainable methods to supplement traditional blood culture-based surveillance systems are highly desirable, not only to evaluate the burden of enteric fever but also to aid in prioritization and monitoring the impact of interventions. Recently, the World Health Organization (WHO) adopted a recommendation for use of typhoid conjugate vaccines (TCVs) in settings with a high burden of typhoid and has prequalified the first TCV.^3,4^ Countries will now face important decisions about whether to provide TCVs and at what geographic scale. These decisions should be based on up-to-date, geographically representative data. An environmental, water-based surveillance strategy could help fill this knowledge gap by leveraging the important role that water has in *Salmonella* Typhi/Paratyphi A transmission.^5–8^ If areas with high detectable levels of typhoid in the water supply overlap with areas of typhoid disease, then sampling water could be utilized as a preliminary surveillance proxy that can guide informed selection of geographical locations for blood culture surveillance and assist policy decisions on investing to control typhoid.

There are no established methods to reliably isolate *Salmonella* Typhi/Paratyphi A from water. Recently, a quantitative real-time PCR (qPCR)–based method was used to demonstrate the presence of their DNA in public drinking water sources in Kathmandu, Nepal, a promising step toward this goal. However, all of the water samples from this study were from the same high-burden typhoid community, precluding interpretation of whether *Salmonella* Typhi/Paratyphi A DNA in water can be used to distinguish high from low typhoid burden settings. In addition, some recent qPCR-based studies using blood specimens have shown high rates of detection of pathogenic DNA in both cases and healthy controls, making the interpretation of PCR-positive samples problematic (e.g. the Pneumonia Etiology Research for Child Health, and the Aetiology of Neonatal Infections in South Asia studies^9^). Therefore, to validate this novel PCR approach as a tool for supplemental enteric fever surveillance using water samples, we aimed to test this method in well-characterized high- and very low–burden settings in Bangladesh.

Enteric fever burden in Bangladesh is high in urban areas.^8,9^ Since 2012, we have been conducting surveillance to monitor enteric fever, pneumonia, meningitis, and sepsis (supported by the WHO) in two hospitals in urban Dhaka (Dhaka Shishu Hospital [DSH], and Shishu Shasthya Foundation Hospital, [SSFH]), and in one hospital in Mirzapur (Kumudini Women Medical College and Hospital [KMWCH]), a rural district approximately 60 km north of Dhaka.^10^ From January 2016 through December 2017, we performed 19,265 blood cultures in DSH, of which 855 (4.4%) were culture-confirmed for *Salmonella* Typhi/Paratyphi A. By contrast, in Mirzapur, Typhi/Paratyphi A was found in 25 of the 15,455 (0.2%) blood cultures performed. To test whether water sampling could serve as a proxy tool to approximate the community burden of enteric fever, we compared water contamination with typhoidal *Salmonella* DNA in drinking water sources in Dhaka, where the burden of enteric fever is high, and in Mirzapur, where the burden is low.

In urban Dhaka, we selected 59 households of febrile patients attending DSH or SSFH from March to June 2016, whose blood culture yielded the growth of *Salmonella* Typhi/Paratyphi A (Supplemental Table 1). We requested the household members to identify their primary source of drinking water and, after obtaining informed consent, collected water from that source. The sources included running tap (*n* = 18), reserve tank (*n* = 40), or tube well (*n* = 1), most of which provided water to multiple neighboring households/families. In Mirzapur, we collected water from a total of 33 sources in June 2017. There was only one blood culture–positive enteric fever case recorded that month in our surveillance hospital KWMCH, which provides health care to about 50% of the Mirzapur population; we collected two water samples (tube well used for drinking water and pond used for bathing) from the household of this patient. We also collected water from deep tube wells, primary source of drinking water, located in 28 households randomly selected from a population-based demographic surveillance program. Finally, we collected one sample from the supply water reserve tank of a commercial complex in Mirzapur. After collecting 1 L of water from each source in a sterile bottle, each bottle was transported to the laboratory within 3 hours and then stored at 4°C until processing. All samples were processed within 4 days of collection. We recorded the location of each sampling site using a global positioning system tracking device (InReach SE+, Garmin, Lawrence, KS) (Figure 1).

**Figure 1.**
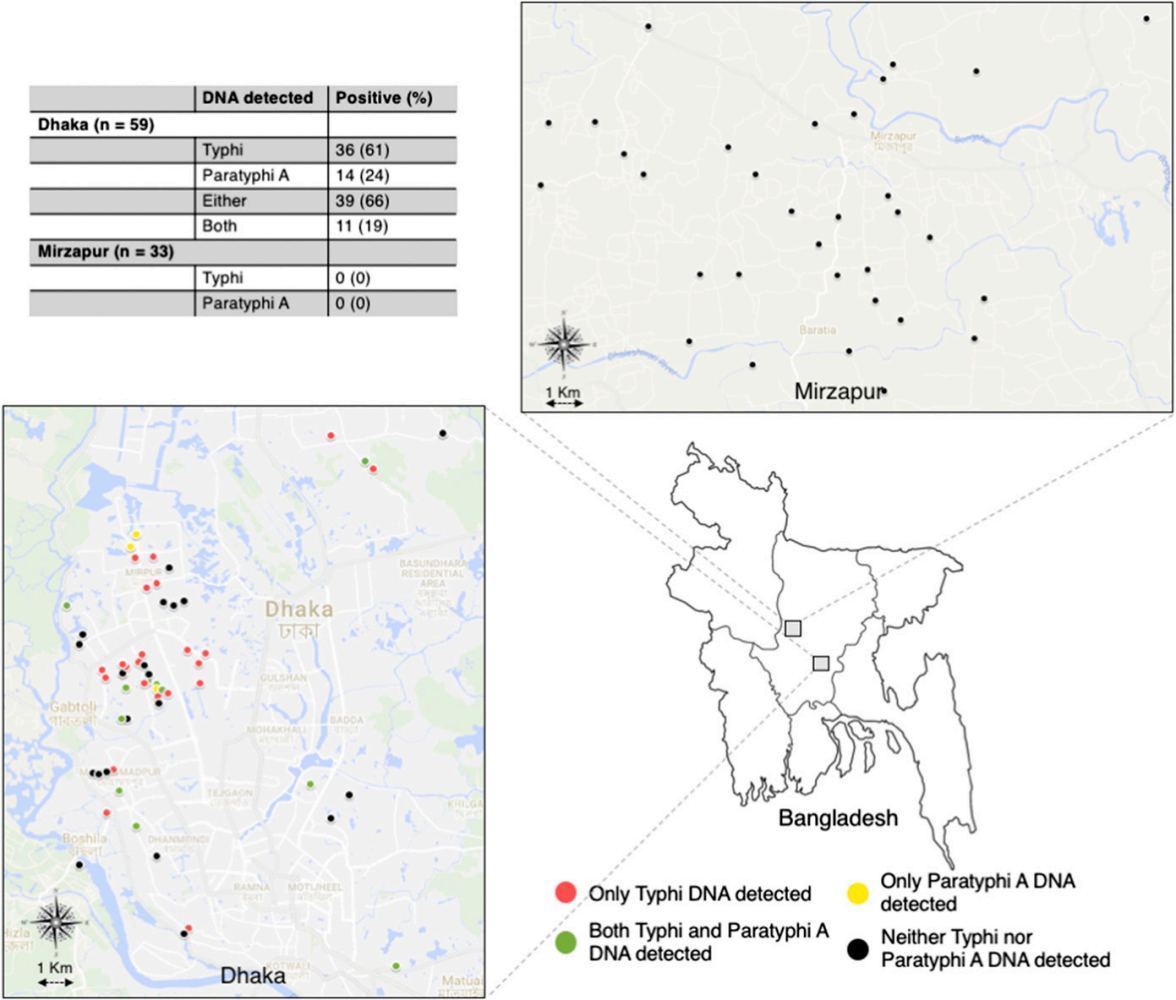
Location of water sample collection and detection of *Salmonella* Typhi and *Salmonella* Paratyphi A DNA in water samples of urban Dhaka and rural Mirzapur, Bangladesh.

Total metagenomic DNA was extracted and used as a template for qPCR amplification to detect *Salmonella* Typhi and Paratyphi A using previously described methods.^11^ Briefly, vacuum filtration was performed using a 0.4 μm Nalgene filtering unit (Thermo Fisher Scientific, Waltham, MA). The filter membrane was removed, cut into pieces, and vigorously washed before DNA extraction (Metagenomic DNA Isolation Kit for water; Epicentre (an Illumina company), Madison, WI); DNA was eluted in 50 μL of Tris-EDTA (TE) buffer. For qPCR amplifications, 25 μL reactions were performed using 2xTaqMan^®^ universal PCR mastermix (Thermo Fischer Scientific) and 4 μL extracted DNA, on Applied Biosystems 7500 Fast Dx platform using the previously described primer and probes.^11,12^ To calculate DNA copy number, we amplified three positive controls of bacterial suspension standards, ranging from 10^−1^ × 0.5 to 10^−3^ × 0.5 McFarland, concurrently with a subset of the samples. A positive reaction in a qPCR assay is detected as a fluorescent signal accumulates, and the number of cycles required to cross a threshold of detection is called the cycle threshold (Ct) value. Ct values of the extracted DNA from water samples and positive controls were calculated. Comparing the Ct values of the extracted DNA and the positive controls, DNA copy number in water sample was calculated using the following formula:

$$C=\frac{\left(S.t.10^{-n}.2^{x_{n}-y}\right)+\left(S.t.10^{1-n}.2^{x_{n-1}-y}\right)+\left(S.t.10^{2-n}.2^{x_{n-2}-y}\right)+\;\ldots +\;\ldots }{n}\;\times \;\frac{E}{t}$$

where*C* = Copy number in water sample DNA.*n* = Number of control DNA dilutions.*S* = Initial concentration of positive control DNA (concentration of 0.5 McFarland = 1.5 × 10^8^ cfu/mL).*t* = DNA template volume used in qPCR reactions.*x*_*n*_, *x*_*n* − 1_, *x*_*n* − 2_ = Ct values of positive control DNA at different dilutions.*y* = Ct value of sample DNA.*E* = Final elution volume (after DNA extraction [corresponds to 1,000 mL filtered water]).

In the Dhaka water samples, we detected *Salmonella* Typhi and/or Paratyphi A DNA from 39 of 59 (66%) households (Figure 1). *Salmonella* Typhi DNA was detected in 36 (61%) samples and *Salmonella* Paratyphi A in 14 (23%) samples; only *Salmonella* Typhi DNA was detected in 25 (42%), only *Salmonella* Paratyphi A in three (5%) and both in 11 (19%) samples. The Ct value of the positive samples ranged from 21 to 39 for *Salmonella* Typhi, which corresponded to median concentration of 559 copies/L (interquartile range, IQR: 154–680). For *Salmonella* Paratyphi A, the range of Ct values was higher, 34 to 37, which, in turn, corresponded to a lower median concentration of 162 copies/L (IQR: 111–587). Neither *Salmonella* Typhi nor Paratyphi A DNA were detected in the 33 water samples from Mirzapur.

These findings are consistent with our blood culture–based surveillance studies and suggest contamination of the water supply of Dhaka city corporation, supporting evidence that the water supply is vital for disseminating these pathogens in Dhaka. Extending into previous work from Nepal*,* which showed a high prevalence of typhoidal *Salmonella* DNA in water sources from endemic areas, we further highlight the potential utility of water sampling as a tool to supplement traditional blood culture–based surveillance for enteric fever.^11^

The results of this study should be interpreted within the context of several limitations. First, in Dhaka, we collected water samples from households of patients with culture-confirmed enteric fever, whereas in Mirzapur, where enteric fever is uncommon, we collected water from a random sample of water sources that were largely representative of the community. We were not able to collect water from households of enteric fever–positive cases in Mirzapur because of its very low burden. However, considering that most water samples were collected from reserve tanks or running taps in Dhaka that provide water to multiple households/families, our data demonstrate the magnitude of exposure to typhoidal *Salmonella* of the population. Second, water samples in Dhaka were collected in 2016 and a year later in Mirzapur, which might affect the positivity rates at the sites. Further work is needed to characterize the relationship between enteric fever incidence and water supply contamination. We intend to conduct a much larger study based on this method to test randomly collected water samples for both Dhaka and Mirzapur in the future. Third, PCR demonstrated the presence of typhoidal *Salmonella* DNA, but did not prove that viable bacteria were present. However, as an epidemiologic tool for assessing disease burden, the presence of DNA from these human-restricted pathogens implies that the organisms were circulating locally. Further research to develop methods to reliably isolate *Salmonella* Typhi and Paratyphi A from water would not only provide further evidence for exposure risk, but also permit whole genome sequence for downstream phylogenetic analysis and the detection of antimicrobial resistance genes, which would improve our understanding of the epidemiology of typhoidal *Salmonella*.

Overall, our findings suggest that water sampling to detect typhoidal *Salmonella* DNA is promising as a low-cost tool to rapidly distinguish high- and low-risk areas for enteric fever. If we find similar results in other typhoid endemic location, then presence or absence of *Salmonella* Typhi/Paratyphi A DNA in water may be applied to map routes of disease transmission and pinpoint sources of supply contamination. This tool may additionally generate community-level data to evaluate the impact of interventions including the introduction of TCV, water improvement projects, and sanitation and hygiene systems.

## Supplementary Material

Supplemental table
