# Supplementary material for: Evaluating PCR-Based Detection of Salmonella Typhi and Paratyphi A in the Environment as an Enteric Fever Surveillance Tool
Source: Am J Trop Med Hyg. 2018 Nov 12;100(1):43–6. doi: 10.4269/ajtmh.18-0428 (PMC6335896; doi:10.4269/ajtmh.18-0428)
Supplement: Supplementary file 1 [file tpmd180428.SD1.pdf]

Supplementary Table S1. Characteristics of water samples collected from Dhaka and Mirzapur in Bangladesh.

Sample Number	Water collection date	Ct, *Salmonella* Typhi	Ct, *Salmonella* Paratyphi A	Water source
Dhaka-01	08-Mar-16	32.82099152	Not detected	Deep Tubewell
Dhaka-02	08-Mar-16	Not detected	36.53629684	Reserve Tank
Dhaka-03	08-Mar-16	35.67969131	Not detected	Direct Tap
Dhaka-04	10-Mar-16	Not detected	Not detected	Direct Tap
Dhaka-05	14-Mar-16	23.48597908	35.00601196	Reserve Tank
Dhaka-06	15-Mar-16	23.48128891	35.56411743	Direct Tap
Dhaka-07	15-Mar-16	21.54895973	35.60522079	Reserve Tank
Dhaka-08	15-Mar-16	Not detected	Not detected	Direct Tap
Dhaka-09	19-Mar-16	28.12397385	36.8869133	Direct Tap
Dhaka-10	19-Mar-16	33.30073929	Not detected	Reserve Tank
Dhaka-11	19-Mar-16	27.40325928	36.82773972	Direct Tap
Dhaka-12	20-Mar-16	33.44520187	36.70759583	Direct Tap
Dhaka-13	21-Mar-16	Not detected	Not detected	Direct Tap
Dhaka-14	21-Mar-16	34.72368622	Not detected	Reserve Tank
Dhaka-15	22-Mar-16	33.04956818	Not detected	Reserve Tank
Dhaka-16	27-Mar-16	34.98424911	Not detected	Reserve Tank
Dhaka-17	22-Mar-16	Not detected	35.61887741	Direct Tap
Dhaka-18	27-Mar-16	34.03388977	Not detected	Direct Tap
Dhaka-19	27-Mar-16	Not detected	Not detected	Reserve Tank
Dhaka-20	29-Mar-16	35.76179123	Not detected	Reserve Tank
Dhaka-21	30-Mar-16	Not detected	Not detected	Reserve Tank
Dhaka-22	30-Mar-16	Not detected	Not detected	Direct Tap
Dhaka-23	31-Mar-16	31.16184616	36.25772095	Direct Tap
Dhaka-24	02-Apr-16	31.4448204	34.38116837	Direct Tap
Dhaka-25	02-Apr-16	33.4496994	36.8572998	Direct Tap
Dhaka-26	10-Apr-16	Not detected	Not detected	Reserve Tank
Dhaka-27	06-Apr-16	33.94907761	Not detected	Reserve Tank
Dhaka-28	09-Apr-16	34.7339592	Not detected	Reserve Tank
Dhaka-29	10-Apr-16	Not detected	Not detected	Direct Tap
Dhaka-30	17-Apr-16	Not detected	37.2097168	Reserve Tank
Dhaka-31	16-Apr-16	Not detected	Not detected	Reserve Tank
Dhaka-32	23-Apr-16	34.54298401	Not detected	Reserve Tank
Dhaka-33	01-Jun-16	38.96130371	Not detected	Reserve Tank

Dhaka-34	07-May-16	34.55870438	Not detected	Reserve Tank
Dhaka-35	07-May-16	Not detected	Not detected	Reserve Tank
Dhaka-36	16-May-16	Not detected	Not detected	Reserve Tank
Dhaka-37	17-May-16	34.95342255	Not detected	Reserve Tank
Dhaka-38	25-May-16	36.81695938	Not detected	Reserve Tank
Dhaka-39	25-May-16	Not detected	Not detected	Reserve Tank
Dhaka-40	12-Jul-16	38.86722946	Not detected	Reserve Tank
Dhaka-41	16-May-16	Not detected	Not detected	Reserve Tank
Dhaka-42	22-May-16	34.06170273	36.68687439	Reserve Tank
Dhaka-43	01-Jun-16	Not detected	Not detected	Reserve Tank
Dhaka-44	31-May-16	Not detected	Not detected	Reserve Tank
Dhaka-45	29-May-16	Not detected	Not detected	Reserve Tank
Dhaka-46	31-May-16	Not detected	Not detected	Direct Tap
Dhaka-47	14-Jun-16	35.73158264	Not detected	Reserve Tank
Dhaka-48	29-May-16	Not detected	Not detected	Reserve Tank
Dhaka-49	01-Jun-16	35.91573334	Not detected	Reserve Tank
Dhaka-50	29-May-16	34.36089325	Not detected	Reserve Tank
Dhaka-51	12-Jul-16	34.39375687	34.68935394	Reserve Tank
Dhaka-52	02-Jun-16	Not detected	Not detected	Reserve Tank
Dhaka-53	02-Jun-16	37.04704285	Not detected	Direct Tap
Dhaka-54	02-Jun-16	Not detected	Not detected	Reserve Tank
Dhaka-55	19-Jun-16	36.34250259	Not detected	Reserve Tank
Dhaka-56	14-Jun-16	33.59719849	Not detected	Direct Tap
Dhaka-57	19-Jun-16	33.66790771	Not detected	Reserve Tank
Dhaka-58	16-Jun-16	34.43291855	Not detected	Reserve Tank
Dhaka-59	16-Jun-16	33.50614548	Not detected	Reserve Tank
Mirzapur-01	01-Jun-17	Not detected	Not detected	Deep Tubewell
Mirzapur-02	01-Jun-17	Not detected	Not detected	Deep Tubewell
Mirzapur-03	01-Jun-17	Not detected	Not detected	Deep Tubewell
Mirzapur-04	01-Jun-17	Not detected	Not detected	Deep Tubewell
Mirzapur-05	01-Jun-17	Not detected	Not detected	Deep Tubewell
Mirzapur-06	01-Jun-17	Not detected	Not detected	Deep Tubewell
Mirzapur-07	01-Jun-17	Not detected	Not detected	Deep Tubewell
Mirzapur-08	01-Jun-17	Not detected	Not detected	Deep Tubewell
Mirzapur-09	01-Jun-17	Not detected	Not detected	Deep Tubewell
Mirzapur-10	01-Jun-17	Not detected	Not detected	Deep Tubewell
Mirzapur-11	03-Jun-17	Not detected	Not detected	Deep Tubewell
Mirzapur-12	03-Jun-17	Not detected	Not detected	Deep Tubewell
Mirzapur-13	03-Jun-17	Not detected	Not detected	Deep Tubewell

Mirzapur-14	03-Jun-17	Not detected	Not detected	Deep Tubewell
Mirzapur-15	03-Jun-17	Not detected	Not detected	Deep Tubewell
Mirzapur-16	04-Jun-17	Not detected	Not detected	Deep Tubewell
Mirzapur-17	04-Jun-17	Not detected	Not detected	Deep Tubewell
Mirzapur-18	04-Jun-17	Not detected	Not detected	Deep Tubewell
Mirzapur-19	04-Jun-17	Not detected	Not detected	Deep Tubewell
Mirzapur-20	04-Jun-17	Not detected	Not detected	Deep Tubewell
Mirzapur-21	04-Jun-17	Not detected	Not detected	Deep Tubewell
Mirzapur-22	04-Jun-17	Not detected	Not detected	Deep Tubewell
Mirzapur-23	04-Jun-17	Not detected	Not detected	Deep Tubewell
Mirzapur-24	05-Jun-17	Not detected	Not detected	Deep Tubewell
Mirzapur-25	05-Jun-17	Not detected	Not detected	Deep Tubewell
Mirzapur-26	05-Jun-17	Not detected	Not detected	Deep Tubewell
Mirzapur-27	05-Jun-17	Not detected	Not detected	Deep Tubewell
Mirzapur-28	05-Jun-17	Not detected	Not detected	Deep Tubewell
Mirzapur-29	05-Jun-17	Not detected	Not detected	Deep Tubewell
Mirzapur-30	05-Jun-17	Not detected	Not detected	Deep Tubewell
Mirzapur-31	11-Jun-17	Not detected	Not detected	Deep Tubewell
Mirzapur-32	11-Jun-17	Not detected	Not detected	Pond
Mirzapur-33	11-Jun-17	Not detected	Not detected	Reserve Tank
